# Assessing the effectiveness of High Intensity Interval Training (HIIT) for smoking cessation in women: HIIT to quit study protocol

**DOI:** 10.1186/s12889-015-2631-3

**Published:** 2015-12-29

**Authors:** Toby G. Pavey, Coral E. Gartner, Jeff S. Coombes, Wendy J. Brown

**Affiliations:** Centre for Research on Exercise, Physical Activity and Health (CRExPAH), School of Human Movement and Nutrition Sciences, The University of Queensland, St. Lucia Campus, Brisbane, QLD, 4072 Australia; School of Public Health, The University of Queensland, Herston Campus, Brisbane, 4006 Australia

**Keywords:** Exercise, Physical activity, Smoking cessation, Women, High intensity

## Abstract

**Background:**

Smoking and physical inactivity are major risk factors for heart disease. Linking strategies that promote improvements in fitness and assist quitting smoking has potential to address both these risk factors simultaneously. The objective of this study is to compare the effects of two exercise interventions (high intensity interval training (HIIT) and lifestyle physical activity) on smoking cessation in female smokers.

**Method/design:**

This study will use a randomised controlled trial design. Participants: Women aged 18–55 years who smoke ≥ 5 cigarettes/day, and want to quit smoking. Intervention: all participants will receive usual care for quitting smoking. Group 1 - will complete two gym-based supervised HIIT sessions/week and one home-based HIIT session/week. At each training session participants will be asked to complete four 4-min (4 **×** 4 min) intervals at approximately 90 % of maximum heart rate interspersed with 3- min recovery periods. Group 2 - participants will receive a resource pack and pedometer, and will be asked to use the 10,000 steps log book to record steps and other physical activities. The aim will be to increase daily steps to 10,000 steps/day. Analysis will be intention to treat and measures will include smoking cessation, withdrawal and cravings, fitness, physical activity, and well-being.

**Discussion:**

The study builds on previous research suggesting that exercise intensity may influence the efficacy of exercise as a smoking cessation intervention. The hypothesis is that HIIT will improve fitness and assist women to quit smoking.

**Australian New Zealand Clinical Trials Registry:**

ACTRN12614001255673 (Registration date 02/12/2014)

## Background

Tobacco smoking is a major cause of cardiovascular disease, chronic obstructive pulmonary disease and many cancers. In women, research has shown that between the ages of 18 to 55 years, the largest population risk for heart disease is attributable to smoking (particularly in young women) and physical inactivity [1]. Despite steady declines in smoking prevalence over the past thirty years, in 2011–12, 16 % (1.4 million) of Australian women aged 18 years and over smoked daily. The 2009 National Preventative Health Strategy calls for a target of 10 % or less for smoking prevalence by 2020. This target will not be reached unless rates of cessation substantially increase [2]. More than half of all smokers want to quit [3], but many struggle to achieve abstinence, and relapse is common, with only 5 % of smokers who quit without any assistance still abstinent at 6 months [4]. As many smokers cite ‘wanting to get fit’ as a motivation for quitting [5], linking strategies that will simultaneously promote improvement in fitness and assist quitting has the potential to change both behaviours simultaneously [6].

There is evidence for a positive effect of exercise when quitting smoking [7], with physical activity (PA) reducing cigarette cravings and withdrawal symptoms [8]. However, the evidence for the efficacy of PA as an aid to quitting smoking is mixed. A recent systematic review of 15 randomised controlled trials highlighted insufficient exercise intensity as a potential limitation of interventions tested in previous trials which did not improve smoking abstinence rates [7].

High Intensity Interval Training (HIIT) could overcome the potential limitation of insufficient intensity, by providing alternating short bursts of high intensity exercise with recovery periods of light exercise. HIIT provides rapid physiological adaptations, as indicated by improvements in maximal oxygen uptake (VO_2_max), anaerobic threshold and stroke volume [9, 10].

### Aims

The aims of this project are to: (1) compare the effects of two exercise interventions (HIIT and a ’10,000 steps’ pedometer based intervention) combined with usual care smoking cessation support, on cessation rates in female smokers who wish to quit; (2) assess the effects of HIIT and 10,000 steps interventions on weight change and (3) assess whether improvements in fitness, vitality and intrinsic motivation are mediators of the relationship between activity and smoking cessation.

## Methods

### Design, participants and recruitment

**Fig. 1:**
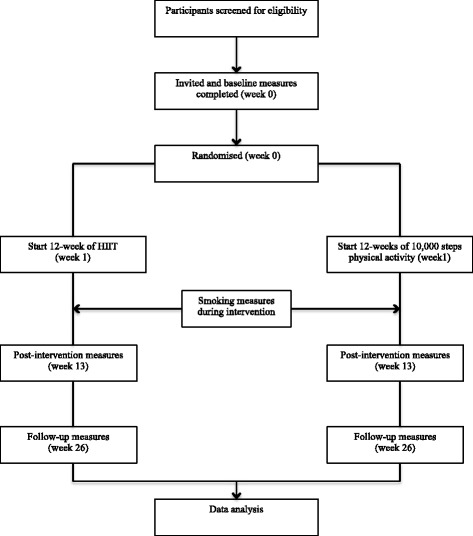
Flow of study

The study has received ethical approval from the Medical Research Ethics Committee of The University of Queensland (#2014001266). This study will use a two group randomised controlled trial design. Inclusion and exclusion criteria are provided in Table 1. Women aged 18–55, smoking at least five cigarettes a day, and who identify as being willing and ready to make a quit attempt will be recruited. Recruitment will be from the local population using radio and print media, advertising, and through collaborations with local health services. A phone number, study email and website will be available for interested participants to contact the research team. A member of the research team will follow-up with a phone call to screen for eligibility, explain the main requirements of the study, and send the Participant Information and Consent Form (PICF). If women wish to participate they will be invited to attend a screening/baseline visit (Fig. 1) and sign the PICF before any data are collected.

**Table 1:** Inclusion/exclusion criteria

Inclusion criteria	Exclusion criteria
Women aged 18 to 55 years	Currently using pharmacological quit smoking aids
Currently smoking at least 5 cigarettes per day	Medical problems that increase risk of adverse events during exercise (including unstable angina, pulmonary disease, uncontrolled hypertension, cardiomyopathty, orthopaedic or neurological limitations)
Desire to quit smoking	Planned surgery during research period
Willing to attend the gym twice weekly if allocated to HIIT intervention group	Current or planned pregnancy
	Drug or alcohol abuse

**Fig. 2:**
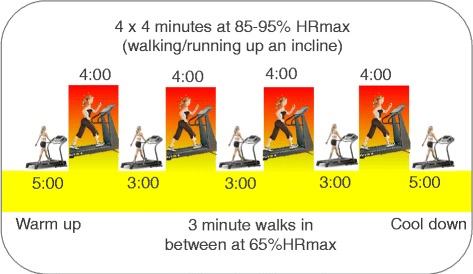
Example of a 35 min 4 × 4 HIIT session

### Randomisation, allocation and concealment

Participants who complete baseline measures will be randomised into one of two groups: 1) HIIT or 2) 10,000 steps (Fig. 1). Each participant will be randomized into one of the two study conditions according to an allocation sequence generated by a computer program and concealed within sealed opaque sequentially numbered envelopes. Analyses will adjust for any baseline differences, consistent with standard procedures. Participants will be allocated to conditions one-at-a-time, allowing for a “rolling start” to the intervention.

### Intervention

*Usual care cessation support:* Participants in both groups will receive usual care smoking cessation support, based on the Smoking Cessation Guidelines for Australian General Practice [11] This includes a quit pack (available from Quitline), which covers planning and preparing to quit, strategies for quit success, and coping with cravings. Participants will also be directed to other resources, including the ‘My QuitBuddy’ mobile app, and the web-based QuitCoach. These resources provide personalized quit plans, monitor progress and money saved, and motivation and support from others using the app. Participants will also be directed to call Quitline should they wish to speak to a counsellor about their quit attempt. Quitline is a confidential service with a professional advisor available for counseling and advice on quitting smoking.

*10,000 steps pedometer group:* participants assigned to this group will receive a resource pack and pedometer, and will be asked to increase their daily steps to 10,000. The resource pack contains a log-book to record steps/day, number of cigarettes smoked, and hints and tips for increasing step counts. The log-book is based on resources used by the Queensland 10,000 steps project (www.10000steps.org.au) Trained research staff will phone these participants once a week to check and record pedometer use and discuss barriers and strategies for successfully increasing PA.

*HIIT group:* participants assigned to this group will complete two gym-based supervised sessions (supervised by trained research staff) and one home-based HIIT session each week. The HIIT protocol will be completed by fast walking or running on a treadmill with the deck inclined to reach the desired intensity, or on a cycle ergometer if individuals are unable or do not wish to use the treadmill. The 35 min protocol consists of: warm-up at 65 % of heart rate maximum (HRmax) (5 min); 4 **×** 4 min intervals at 85–95 % HRmax (with the target zone reached by at least two minutes), interspersed with 3 min recovery at 65 % HRmax; and cool down (5 min), with a total exercise time of 35 min (see Fig. 2). Attendance at supervised sessions and adherence to home-based sessions (including compliance with target heart rate) will be monitored and recorded by research staff.

After the 12-week intervention researcher support will be removed from both groups (e.g. removal of supervised HIIT sessions and 10,000 steps support). Researcher contact will only be made to arrange and complete follow-up measures during weeks 13 and 26.

### Data collection

Participants will be assessed on three occasions: at baseline, week 13 and week 26 (see Fig. 1). Cigarettes per day will be measured weekly. Withdrawal symptoms, cigarette cravings and feelings of stress, will be monitored during the intervention. See Table 2 for full list of measures, and measurement time-points. Exercise and fitness measures will be completed by research staff whom will be blinded to participant group allocation.

**Table 2:** Timeline for data collection

	Measurement time-point					
Measures	Baseline	Week 2	Week 4	Week 8	Week 13	Week 26
Cigarettes per day (weekly)						
Smoking cessation					X	X
Withdrawal symptoms	X	X	X	X	X	X
Cigarette cravings	X	X	X	X	X	X
Cigarette dependence	X				X	X
Subjective stress	X		X	X	X	X
Saliva cotinine	X				X	X
Cardiorespiratory fitness	X				X	X
Lung function	X				X	X
Body composition	X				X	X
Physical activity	X				X	X
Well-being	X				X	X
Motivation	X				X	X
Demographic information	X				X	X

### Smoking measures

*Smoking abstinence and cessation* will be assessed using the Russell Standard [12]. This includes self-reported ‘quitting’ (not smoking for two weeks) and expelled carbon monoxide concentration (Micro Smokerlyser; Pico simple, Harrietsham, UK) of less than 10 ppm (which reflects abstinence from smoking during the previous 24-h). *Saliva* will be collected by drooling into a 50 ml Falcon tube. The sample will be frozen and stored for analysis of cotinine, which is a metabolite of nicotine. *Self-reported number of cigarettes* smoked will be recorded each week. *Withdrawal symptoms* and *cravings* will be assessed using the Mood and Physical Symptoms Scale (MPSS) [13]. *Smoking dependency* will be assessed using the Fagerström Test for Nicotine Dependance (FTND) [14]. *Subjective stress* will be assessed using the 4-item Perceived Stress Scale (PSS) to monitor any changes associated with smoking reduction or increasing PA [15].

### Exercise, fitness and physical activity measures

*Cardiorespiratory fitness (VO2max)* will be assessed using a graded exercise test to exhaustion. We will analyse expired air using a metabolic system (Trueone 2400, Sandy, USA) to calculate the maximal rate of oxygen consumed and used during the test. We will monitor participants’ ECG (CASE, Milwaukee, USA) during and after the test. Maximal heart rate attained will be used to determine the training intensity for each participant. We will measure blood pressure prior to and during the test.

This fitness test will be completed on a treadmill (T2100, Little Chalfont, UK) or a cycle ergometer (Lode Excalibur Sport, Groningen, Netherlands) if individuals are unable or do not wish to use the treadmill. The treadmill test will use a ramp protocol where the inclination is constant (5.5 %) and the speed increased 0.5 km/h every minute, starting at 4 km/h. The cycle protocol includes a 2 **×** 4 min warm-up at 25 W and 50 W. The test starts at 50 W and increases by 25 W every minute. We will ask participants to refrain from smoking two hours before the test, and from drinking alcohol, or doing any vigorous or moderate intensity activities 12-h before the test.

*Lung function* will be assessed by forced vital capacity [FVC], forced expiratory volume in 1 s [FEV_1_] and FEV_1_/FVC ratio, using a Vitalograph (2150, Ennis, Ireland). We will ask participants to inhale completely and rapidly through the mouth (not the nose), then exhale maximally and forcefully, with their lips creating an airtight seal around the mouthpiece, until no more air can be expelled (minimum of six seconds). Participants will repeat the manoeuvre three times, with a rest period of ≥1 min between efforts. Peak expiratory flow rate [PEFR]) will be assessed using a portable peak flow meter (Assess, Cedar Grove, USA), using the same technique as described above. Participants will repeat the manoeuvre three times, with a rest period of ≥1 min between efforts.

*Body composition* will be assessed by researcher measured body mass index (height, weight), and waist circumference. Standing height and weight will be measured using a stadiometer (SECA 217-172-1009, Hamburg, Germany) and electronic scale (Charder MS 3200, Hamburg, Germany) respectively. Waist circumference will be measured using a tape passed around the narrowest point of the abdomen. We will take each measure twice and use the average measure obtained, unless the first and second measures vary by more than 1 %, in which case we will use the median of three measurements. Body mass index will be calculated as weight (kg)/height^2^ (m^2^).

*Physical activity and sedentary time* will be measured using a wrist-worn GENEActiv accelerometer, which will be worn for one week at each measurement time. The GENEActiv is a tri-axial, ± 6 g seismic acceleration sensor. It is small (36x30x12 cm), lightweight (16 g), waterproof, and offers a near body temperature sensor to help improve the confirmation of wear and non-time. GENEActivs will be configured with a sampling frequency of 80 Hz, with data uploaded and converted to 15 s epoch .csv files using GENEActiv PC software version 2.1. We will import the epoch files into custom built Excel spreadsheets that will compute the most likely posture (lying, sitting, standing, ambulation), activity intensity (sedentary, light, moderate, vigorous) and sleep. This will provide information on average daily sedentary, active and sleep patterning and behaviour, and will provide a measure of overall weekly physical activity. GENEActiv validity studies have demonstrated strong correlations for criterion validity (r = 0.79 to 0.98) for physical activity and sedentary behaviour [5, 6]. Data in this study will be based on standard criteria for wear time [7].

### Demographic information

Participants will complete a baseline survey with relevant repeated questions at weeks 13 and 26. The surveys will include questions on age, education, occupation, income, health history, and smoking history.

### Self-reported health and behaviour

*Well-being* will be measured with the SF36 (physical and mental summary scores and eight subscales (including vitality) [16]. *Motivation* will be assessed with the Behavioural Regulation in Exercise Questionnaire (BREQ-2) [17].

### Data analysis

The aim of this study is to obtain data on the effects of HIIT training on smoking cessation and intermediate outcomes, such as cigarette cravings and withdrawal symptoms. Using effect size estimates based on results of a previous study using vigorous PA for smoking cessation in women (i.e. OR = 2.09, [18]), adopting a two tailed alpha level of 0.05 and power 0.80, a sample size of 100 will be required. Based on funding and time constraints, the study aims to recruit 50 participants per group. Descriptive statistics will be used to summarize the characteristics of participants in each group, and to describe those who successfully quit. Yates’ corrected Chi-squared test will be used to analyse the difference in the proportions who quit in each group, and changes in categorical (e.g. smoking abstinence) and continuous (e.g. number of cigarettes smoked) variables will be assessed using generalised linear modeling.

## Discussion

Physical inactivity and tobacco smoking are important contributors to the overall burden of disease in Australia, and are significant risk factors for almost all Australia’s major non-communicable physical and mental health problems.

This study is innovative, as it will build on suggestions from previous studies that intensity of exercise may be a critical factor in determining the efficacy of PA interventions for smoking cessation. Our study addresses this issue by comparing the effect of two PA interventions of varying intensity on smoking cessation outcomes.

In examining the notion that HIIT (combined with a quit program) will result in higher quit rates than a more moderate PA intervention, we will also examine novel mediators (fitness, vitality and intrinsic motivation) which will improve understanding of the relationships between HIIT and smoking cessation.

Further, we will assess the effect of exercise intensity on other indicators of smoking, such as withdrawal symptoms, cigarette cravings and weight gain, as these factors are important barriers to smoking cessation. This project will address the weaknesses of previous studies (e.g. poor assessment of intensity, adherence or compliance to exercise) and is novel in design and conceptualisation.

HIIT has potential to impact on individual cardiovascular health and to be a very cost effective intervention, compared with other cardiovascular treatments (e.g. statins), as it may address two major contributors to cardiovascular disease (smoking and physical inactivity) in one intervention. The research will inform efforts to develop novel, innovative, and time efficient strategies for promoting cardiovascular health in women.

## Abbreviations

BREQ: Behavioural Regulation in Exercise Questionnaire
FTND: Fagerström Test for Nicotine Dependance
HIIT: high intensity interval training
HRmax: heart rate maximum
MPSS: Mood and Physical Symptoms Scale
PA: physical activity
PICF: participant information and consent form
PSS: Perceived Stress Scale
VO_2_max: maximal oxygen uptake
